# Acute intratracheal *Pseudomonas aeruginosa *infection in cystic fibrosis mice is age-independent

**DOI:** 10.1186/1465-9921-12-148

**Published:** 2011-11-07

**Authors:** Antje Munder, Florian Wölbeling, Tanja Kerber-Momot, Dirk Wedekind, Ulrich Baumann, Erich Gulbins, Burkhard Tümmler

**Affiliations:** 1 Clinical Research Group, Clinic for Pediatric Pneumology, Allergology and Neonatology, OE 6710, Hannover Medical School, Hannover, Germany; 2 Institute for Functional and Applied Anatomy, OE 4120, Hannover Medical School, Hannover, Germany; 3 Institute of Laboratory Animal Science, OE 8600, Hannover Medical School, Hannover, Germany; 4 Pediatric Pulmonology, Allergology and Neonatology, OE 6710, Hannover Medical School, Hannover, Germany; 5 Department of Molecular Biology, University of Duisburg-Essen, Essen, Germany

**Keywords:** Cystic fibrosis mouse models, intranasal, intratracheal, experimental lung infection, age effect

## Abstract

**Background:**

Since the discovery of the human *CFTR *gene in 1989 various mouse models for cystic fibrosis (CF) have been generated and used as a very suitable and popular tool to approach research on this life-threatening disease. Age related changes regarding the course of disease and susceptibility towards pulmonary infections have been discussed in numerous studies.

**Methods:**

Here, we investigated *Cftr^TgH(neoim)Hgu ^*and *Cftr^tm1Unc^*-Tg*(FABPCFTR)*1Jaw/J CF mice and their non-CF littermates during an acute lung infection with *Pseudomonas aeruginosa *for age dependent effects of their lung function and immune response.

Mice younger than three or older than six months were intratracheally infected with *P. aeruginosa *TBCF10839. The infection was monitored by lung function of the animals using non-invasive head-out spirometry and the time course of physiological parameters over 192 hours. Quantitative bacteriology and lung histopathology of a subgroup of animals were used as endpoint parameters.

**Results:**

Age-dependent changes in lung function and characteristic features for CF like a shallower, faster breathing pattern were observed in both CF mouse models in uninfected state. In contrast infected CF mice did not significantly differ from their non-CF littermates in susceptibility and severity of lung infection in both mouse models and age groups. The transgenic *Cftr^tm1Unc^*-Tg*(FABPCFTR)*1Jaw/J and their non-CF littermates showed a milder course of infection than the *Cftr^TgH(neoim)Hgu ^*CF and their congenic C57Bl/6J non-CF mice suggesting that the genetic background was more important for outcome than *Cftr *dysfunction.

**Conclusions:**

Previous investigations of the same mouse lines have shown a higher airway susceptibility of older CF mice to intranasally applied *P. aeruginosa*. The different outcome of intranasal and intratracheal instillation of bacteria implies that infected CF epithelium is impaired during the initial colonization of upper airways, but not in the subsequent response of host defense.

## Background

Cystic fibrosis (CF) is the most common life-shortening autosomal recessive disease within the Caucasian population and is caused by mutations in the *Cystic Fibrosis Transmembrane Conductance Regulator *(*CFTR*) gene [1]. The CFTR protein functions as a cAMP-regulated chloride channel in the apical membrane of epithelial cells. The symptoms of CF are caused by an impaired function of exocrine glands in many CFTR expressing organs, predominantly within the gastrointestinal and respiratory tracts. In most cases the progressive decrease of lung function is life limiting for CF patients. In this context, the opportunistic bacterial pathogen *Pseudomonas aeruginosa *most commonly causes chronic microbial lung infections, leading to excessive lung tissue remodelling and destruction [2,3]. The bacteria are able to survive in the anaerobic environment of the CF lung [4] and become extremely resistant to the eradication of biofilms in the conducting airways by antibiotic treatment.

In 1989 the coding gene for the CF disease was identified on the long arm of chromosome 7 [5-7], a finding which highly advanced our understanding of CF cell biology and pathophysiology. In 1991 Tata et al. [8] and Yorifuji et al. [9] described the cloning and sequencing of the murine *Cftr *gene which is located in a conserved segment of chromosome 6 and shows 78% amino acid sequence homology to the human *CFTR *gene. Since no spontaneous mutations were known for the murine *Cftr*, different CF mouse models were generated by targeted mutagenesis [10]. Most of these models show massive pathological changes in the intestine, but fail to develop a lung disease comparable to human CF subjects. The reason therefore may due to the short life expectancy of mice or their ability to use alternative chloride channels in the lung epithelium [11-15]. In 1992 Dorin and her coworkers described the *Cftr^TgH(neoim)Hgu ^*mouse which only showed mild gastrointestinal complications, a good survival after weaning and benign respiratory symptoms [16,17]. Another well described and often used mouse model is represented by the transgenic STOCK *Cftr^tm1Unc^*-Tg*(FABPCFTR)*1Jaw/J mouse, which expresses human *CFTR *in the gut under control of the *FABP1 *promoter (fatty acid binding protein1), which prevents it from acute intestinal obstruction [18].

Both CF models were included in a study by Teichgräber et al. [19] in which they detected ceramide accumulation in the murine respiratory epithelium and hypothesized that this accumulation leads to inflammation and cell death and increases infection susceptibility towards *P. aeruginosa *in CF patients. A significant increase of ceramide in the lung epithelium of both CF models was found to be associated with higher bacterial numbers, an accumulation of neutrophils and alveolar macrophages and increased cell death. All effects became more prominent with increasing age and started to become visible by around week 16 of murine life. These findings are also consistent with previously published data from P. Durie and associates, who identified many pathological changes in aged CF mice [20].

In this study described here we tested the influence of the described age-dependent ceramide accumulation [21] in our well established mouse model on airway infection with *P. aeruginosa *[22] using young and old mice and identical mouse strains but a different, namely intratracheal infection route. In Teichgräber's study [19] the bacteria were inoculated by intranasal instillation thus targeting both the upper and lower airways. In contrast intratracheal instillation [23] bypasses the upper airways and delivers more bacteria into distal bronchi than the intranasal inoculation. Thus the two infection routes target overlapping, but not matching airway compartments. We monitored the course of infection over 192 hours via several physiological parameters supported by non invasive head-out spirometry and employed quantitative bacteriology and lung histopathology as endpoint parameters. To make a clear distinction we categorized mice in groups younger than three and older than six months. Moreover, we compared the lung function of the CF mice in the uninfected state. The differential outcome of the infection experiments in Teichgräber's and our study led to the conclusion that older CF mice are impaired in their first defence of bacterial clearance, but that otherwise the clinical course of the acute *P. aeruginosa *lung infection is indistinguishable between CF and non-CF mice that share the same genetic background.

## Methods

### Mouse strains

Infection experiments were performed with two CF mouse models (a) B6.129P2(CF/3)-*Cftr^TgH(neoim)Hgu^*, (b) *Cftr^tm1Unc^-*Tg*(FABPCFTR)*1Jaw/J and their respective littermates. According to the nomenclature of Teichgräber et al. the mouse lines are called (a) *Cftr^MHH ^*and (b) *Cftr^KO^*, their non-CF littermates (a) B6 and (b) WT, respectively. In *Cftr^TgH(neoim)Hgu ^*mice the exon 10 of the *Cftr *gene had been disrupted by the insertion of the vector pMCIneoPolyA [16]. Since those mice produced low levels of Cftr [17] but showed a mixed genetic background [24], from the original *Cftr^TgH(neoim)Hgu ^*mutant mouse, CF strain CF/3-*Cftr^TgH(neoim)Hgu ^*was established at the Institute of Laboratory Animal Science of the Hannover Medical School by brother-sister mating for more than 40 generations. Next, the congenic mouse inbred strain B6.129P2(CF/3)- *Cftr^TgH(neoim)Hgu^*, which is used in this study, was generated by 40 backcross generations using CF/3-*Cftr^TgH(neoim)Hgu ^*as donor strain and C57BL/6J as recipient strain [25]. Following the nomenclature of Teichgräber et al. [19] this strain is called *Cftr^MHH^*, syngenic C57BL6/J mice are called B6 and served as controls. *Cftr^MHH ^*mice are regulary monitored for their congenic C57BL/6J status using 27 SNP markers and integrity of the mutant *Cftr *locus by intragenic microsatellite markers [24,26,27].

STOCK *Cftr^tm1Unc^-*Tg*(FABPCFTR)*1Jaw/J mice were obtained from the Jackson Laboratories. These mice, in the following called *Cftr^KO^*, which are of a mixed genetic background consisting of C57BL/6, FVB/N and 129, are knock-outs for the murine *Cftr *gene, but express human CFTR in the gut under control of the *FABP1 *(fatty acid binding protein1) promoter, which prevents acute intestinal obstruction 1 [12,18]. Mice were obtained homozygous and heterozygous for the *Cftr^tm1Unc ^*targeted mutation (tm/tm and tm/+) as well as homozygous and hemizygous for the *FABP-hCFTR *transgene (tg/tg and tg/0). Tm/+ tg/0 mice were used as parents to generate wildtype control mice (called WT). Genotyping was performed using the protocols provided by the JAX lab [28].

Mice were maintained at the Central Animal Facility of the Hannover Medical School, Carl-Neuberg-Str. 1, 30625 Hannover, Germany. They were held in groups of three to five animals animals in microisolator cages (910 cm^2^) with filter top lids and free access to sterilised standard laboratory chow (diet No. 1324, Altromin, Lippe, Germany) and autoclaved, acidulated water at 21 ± 2°C, 55 ± 5% humidity and a 10:14 light-dark-cycle. None of the CF mice showed gastrointestinal complications which would require a special diet. All mice were regularly monitored for infection by typical pathogens according to the FELASA recommendations [29]. All procedures performed on mice were approved by the local district governments (AZ. 33.9-42502-04-08/1528) and carried out according to the ILAR guidelines for the care and use of laboratory animals [30]. Experimental groups of mice were allocated by age of either younger than three (henceforth designated as young) or elder than six months (henceforth designated as old). This selection aimed to mimic the categories of Teichgräber et al. [19] that mice older than 4 months were susceptible to an infection with *P. aeruginosa *whereas the younger mice were more or less resistant.

Due to breeding limitations B6 mice showed a strong predominance of males and no 192 h value of old B6 exist.

### Spirometry

Non-invasive head-out spirometry with 14 parameters was performed on conscious restrained mice [22]. In brief, four mice were investigated in parallel by placing them in glass inserts with their heads protruding out through a set of membranes ensuring an airtight fit. Respiration caused air to flow through a pneumotachograph positioned above the thorax of the animals. The airflow was digitalized and analyzed with the Notocord Hem 4.2.0.241 software package (Notocord Systems SAS, Croissy Sur Seine, France).

### Bacteria

*Pseudomonas aeruginosa *strain TBCF10839 [31] was grown in Luria broth (LB) overnight at 37°C. The overnight culture was washed twice with the same volume of sterile PBS to remove cell detritus and secreted exopolysaccharides, then the optical density of the bacterial suspension was determined and the intended number of colony forming units (CFU) was extrapolated from a standard growth curve. Inocula with 6.0 × 10^5 ^CFU in 30 μl were prepared by dilution with sterile PBS. This infection dose is approximately one tenth of the LD50 of strain TBCF10839 for C57BL/6J mice and was able to produce a clinical infection without mortality.

### Infection protocol

Mice were infected intratracheally (i.t.) with 6.0 × 10^5 ^CFU of *P. aeruginosa *strain TBCF10839 under a light anaesthesia. For detailed description of the view-controlled intratracheal instillation see Munder et al. [23]. To characterize the course of the bacterial infection, the body condition, weight, rectal temperature and lung function of the mice were evaluated as described previously [32]. In brief the overall health of the animals was assessed by vocalisation, piloerection, posture, locomotion, breathing, curiosity, nasal secretion, grooming and dehydration. Dysfunctions in single parameters were assessed by zero, one or two points. The overall fitness of the mice was determined by adding the points resulting in the following score of the mouse body condition: unaffected (0-1); slightly affected (2-4); moderately affected (5-7); severely affected (8-10); moribund (≥ 11).

Non-invasive head-out spirometry. First, spirometric values of uninfected animals (B6, WT, *Cftr^KO ^*and Cftr*^MHH ^*with age young: < 99 days and old: > 179 days) were averaged (median) from three independent measurements preformed on consecutive days. Prior measurements assured that the mice had adapted to the procedure. Lung function measurements of infection experiments were taken daily at the five days prior to inoculation and at time points 4, 6, 8, 10, 12, 18, 24, 48, 72, 96, 120, 144, 168, 192 hours post inoculation.

Forty-eight hours after challenge subgroups of mice were euthanized. Their left lungs were taken for the determination of bacterial counts and the right lungs were stained and examined histologically.

### Pathohistology of the lungs

The right lungs were fixed via the trachea (4% paraformaldehyd), embedded in paraffin and stained with haematoxilin/eosin. One section was selected that showed aspects from all three lobes of the right lung. This slide was examined in twenty fields of view at a 100 fold magnification using a Zeiss Axiophot photomicroscope. Inflammation was assessed using a semi quantitative pathohistological score [22]. Shortly, lung histological changes were scored on a scale from one to three points. Points were given separately for lung parenchyma, airways and lung vessels. The total score classified airway inflammation into the categories: almost not visible (0-5); slight (6-20) moderate (21-40); severe/profound inflammation (41-60). In the current study no more than a medium-grade inflammation was seen, appearing as a purulent alveolar pneumonia with peribronchiolar and perivascular inflammatory infiltrates.

### Lung bacterial numbers

The left lungs of the euthanized mice were ligated, resected and homogenised. Aliquots were plated and bacterial numbers of whole organs were calculated. Previous experiments showed that the distribution of bacteria is approximately equal in left and right lungs after i.t. application (data not shown).

### Statistics

Each CF mouse model and its wild type controls were investigated separately by age group using non-parametric test statistics of SPSS 16 (Version 16.0.2, SPSS Inc, Chicago, USA). p-values (p < 0.05) with subsequent Bonferroni correction were calculated by 2-sided Monte Carlo simulations (100,000 simulations). Hereby groups were composed of equal numbers of mice (perfect match approach).

## Results

### Lung function of CF mice

**Figure 1:**
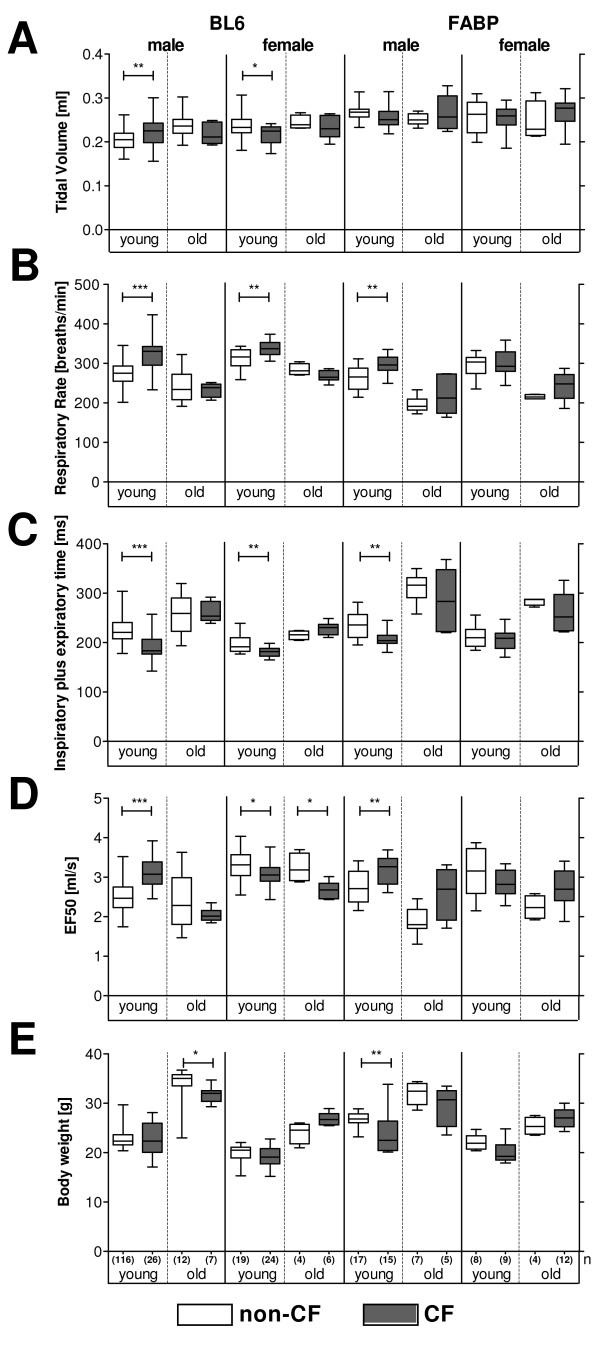
**Respiratory parameters and body weight in non-infected CF mice and their non-CF littermates (left panels: B6, *Cftr^MHH^*; right panels: WT, *Cftr^KO^*)**. At the day of assessment young mice were younger than 100 days and old mice elder than 179 days. The boxplots show the median, inner quartiles and range of the parameters and are clear for wildtype mice (B6, WT) and are shaded in grey for CF mice (*Cftr^MHH^*, *Cftr^KO^*). CF mice and non-CF littermates were compared in their lung function and body weight by Mann-Whitney rank tests (* = p < 0, 05; ** = p < 0, 01; *** = p < 0, 001). The number of investigated animals per group (n) is indicated in Figure 1E. Figure 1A shows that the tidal volume is very similar for all genotypes. The respiratory rate is higher (Figure 1B) and the time for one breath is shorter in CF mice (Figure 1C).

To evaluate the impact of age, genetic background and the *Cftr *mutant construct on respiratory health, we determined lung function in young and old CF mice and their congenic wild-type littermates (Figure 1).

Tidal volume increased with age of the animals reflecting an increase in body mass. Respiration decreased, also characterized by increasing times for one breath. The slower breathing was also characterized by a decrease in the flows of expiration and inspiration.

Body weight measurements showed that *Cftr^KO ^*and WT mice were slightly larger and heavier than *Cftr^MHH ^*and B6 mice. This is mirrored in a slightly higher tidal volume for the *Cftr^KO ^*and WT mice.

All mice irrespective of genotype or gender had a comparable total lung volume (tidal volume, Figure 1A). CF mice, however, achieved the comparable lung volume through an increase in respiratory rate (Figure 1B). Correspondingly the time for one breath (Time of inspiration plus expiration, Figure 1C) was smaller in CF mice. The higher respiratory rate was associated with higher flows as depicted by the EF50 parameter (Midtidal expiratory flow at 50% expiration, Figure 1D).

### Outline of infection experiments

**Figure 2:**
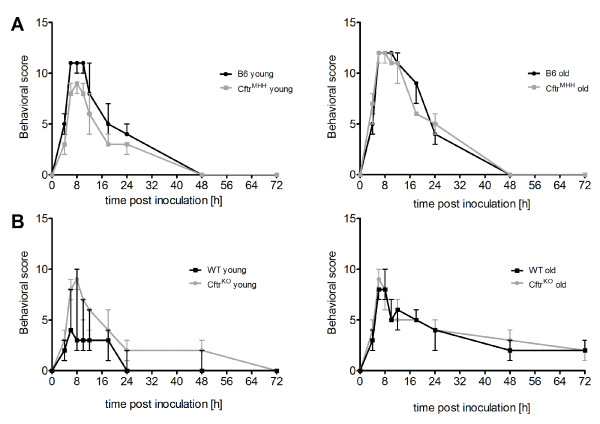
**Time course of the global health score during infection**. The behavioral score describes the fitness of mice during infection. Mice with a C57Bl/6J background (A) were more affected than FABP mice (B). The responses of congenic non-CF and CF mice were indistinguishable (black vs. gray lines in all panels) with the single exception that young FABP WT mice were only mildly affected even during the first 24 h after challenge with *P. aeruginosa *(B, left panel).

**Figure 3:**
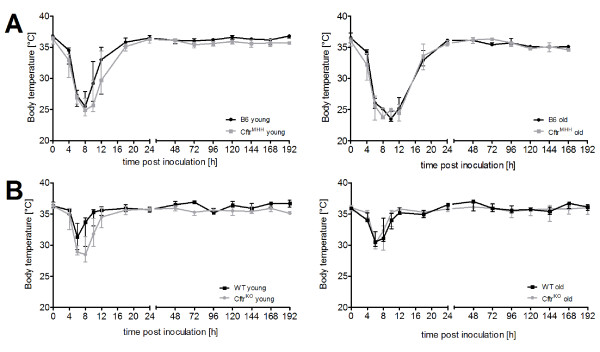
**Time course of rectal body temperature during infection**. Body temperature of all mice showed a strong decrease, which peaked between 6 and 10 hours post inoculation. Temperature declined in C57Bl/6J mouse strains to a minimum of approximately 25°C (A), in FABP mice the minimal temperature was always higher than 30°C (B). Exceptions were the young *Cftr^KO ^*mice with a minimal temperature of 28.5°C at 8 h p.i. In old B6 and *Cftr^MHH ^*mice (A, right panel) temperature remained longer at lower values (until 12 h p.i.), although the recovery to physiological level took the same period of time as in the groups of young *Cftr^MHH ^*and B6 mice (A, left panel. In summary, the C57BL/6J mouse strains showed a stronger depression of body temperature upon airway exposure with *P. aeruginosa *than FABP mice.

**Figure 4:**
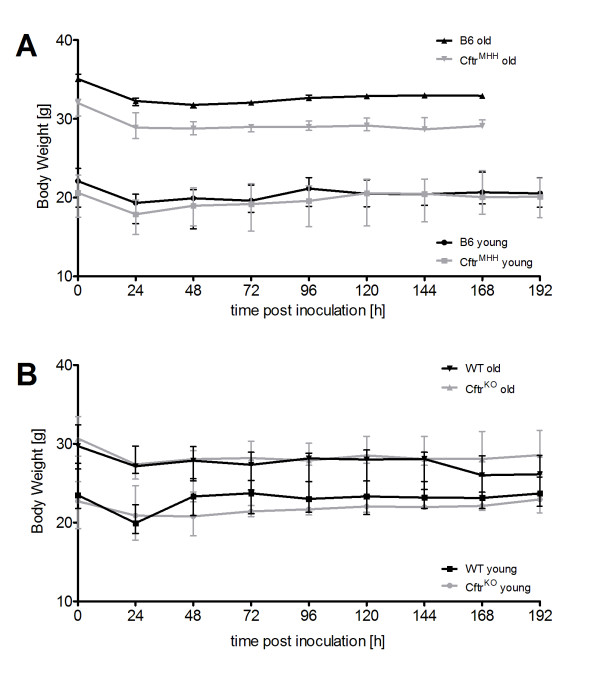
**Time course of body weight during infection**. After challenge with *P. aeruginosa *all animal groups lost weight during the first 24 hours p.i. Young B6 and *Cftr^MHH ^*mice started to regain weight directly thereafter, although this increase was small and both groups had not reached their initial weight by the end of the experiments (A). Weight loss was more pronounced in the old B6 and *Cftr^MHH ^*mice that also did not regain their initial weight by 192 hours p.i. In both CF mouse models CF mice younger than 3 months displayed a reduced body weight compared to their wild type littermates. This tendency became more pronounced in adult C57BL6/J, but not FABP mice thus showing an anthropometric CF phenotype in the former, but not in the latter strain. Please note the different initial values of young and old mice and the stronger impact of gender in the old mice, reflected in the large error bars of old *Cftr^MHH^*, old *Cftr^KO ^*and WT mice (A, B). Since the group of old *Cftr^MHH ^*mice was exclusively formed by male animals, less pronounced intragroup differences were noted (A, grey line with triangles).

**Figure 5:**
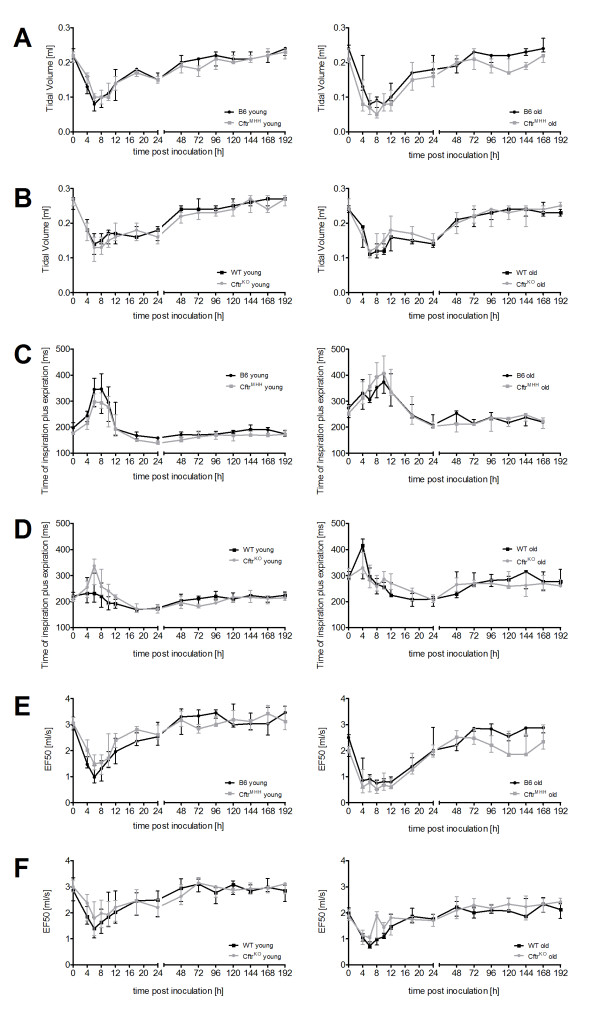
**Time course of selected lung function parameters during infection**. Spirometric curves depicting the time course of Tidal Volume (Total volume inspired and expired in one breath) (A, B), Inspiratory plus Expiratory time (Time required for one breath) (C, D) and Expiratory flow at 50% Expiration (EF50) (E, F) of *Cftr^MHH ^*mice and *Cftr^KO ^*and wild type mice after the intratracheal instillation of *P. aeruginosa*. There are no marked differences for none of the parameters between CF and wild type mice of either genotype. C57BL6/J mice responded stronger to the bacterial infection than FABP mice as indicated by the larger decrease in lung volume (A). As young mice breathe faster than old mice, the overall time for one breath increases with age (C, D).

To monitor the outcome of an intratracheal instillation of *P. aeruginosa *in our CF mouse models and their non-CF littermates, mice of both genders were exactly matched with their corresponding wild type controls by sex, age and body weight. Mice were characterized in their global health score (Figure 2), rectal temperature (Figure 3), body weight (Figure 4) and lung function (Figure 5, Additional file 1) at 14 time points over a period of 192 h.

### Global body condition

B6 mice were more affected by *P. aeruginosa *in their body condition than the *Cftr^KO ^*and their non-CF littermates (Figure 2). With the exception of young *Cftr^KO ^*mice the corresponding CF and non-CF mouse lines exhibited a similar time course of disease symptoms. Animals were notably affected between 6-12 h after inoculation and recovered within the next 48 h. Young WT mice were least affected by the instillation of *P. aeruginosa *into their lungs.

### Rectal body temperature

Upon exposure to bacteria mice did not react with fever but with a drop of body temperature. The temperature profile mimicked the time course of the global health score. *Cftr^MHH ^*and the B6 mice experienced a stronger drop of temperature than the *Cftr^KO ^*and WT mice, the latter being minimally affected with a maximal reduction of rectal temperature of 5°C (Figure 3).

### Body Weight

Within the first 24 h post inoculation the mice lost 8% (old B6) to 13% (young *Cftr^MHH^*) of their initial body weight (Figure 4). By the end of the experiment the young mice had almost completely regained their initial weight, whereas an irreversible weight loss was observed in all old mice. Consistent with an intestinal CF phenotype, old *Cftr^MHH ^*mice were significantly lighter than their congenic B6 mice [24].

### Lung function

The time course during infection is shown exemplarily for tidal volume (the total volume inspired and expired in one breath) (Figure 5A, B), Time of inspiration plus expiration (Figure 5C, D) and EF50 (Figure 5E, F). The data of all 14 measured lung function parameters are shown in the online supplement (Additional file 1). The old non-CF and CF mice showed a similar response in lung function towards the instillation of *P. aeruginosa *into their lungs. Young KO mice differed from their WT FABP littermates in a shorter 'time of pause' prior to and after inoculation, but not in any other of the 14 parameters. In contrast, non-infected young *Cftr^MHH ^*mice showed another breathing pattern than their congenic B6 mice (see above). *Cftr^MHH ^*mice differed from their non-CF congenics in seven lung function parameters with a high respiratory rate as the leading symptom. This characteristic pattern of non-infected *Cftr^MHH ^*mice was also seen in the challenged mice at the late time points of 144, 168 and 192 h when they had recovered from infection. Thus, by the end of experiment the *Cftr^MHH ^*mice had regained the initial lung function phenotype. Besides these CF-genotype-driven differences between congenic mice that were apparently independent of bacterial infection, a differential response of *Cftr^MHH ^*and B6 mice was noted at time points 4 and 6 hours after challenge with *P. aeruginosa*. Lung function slightly, but significantly differed in seven flow or volume parameters (Additional file 1). In summary, differences in lung function between congenic non-CF and CF mice were not detectable or subtle, and if they were more prominent as in the case of *Cftr^MHH ^*mice, they were also existent in non-infected animals.

### Endpoint analyses

**Figure 6:**
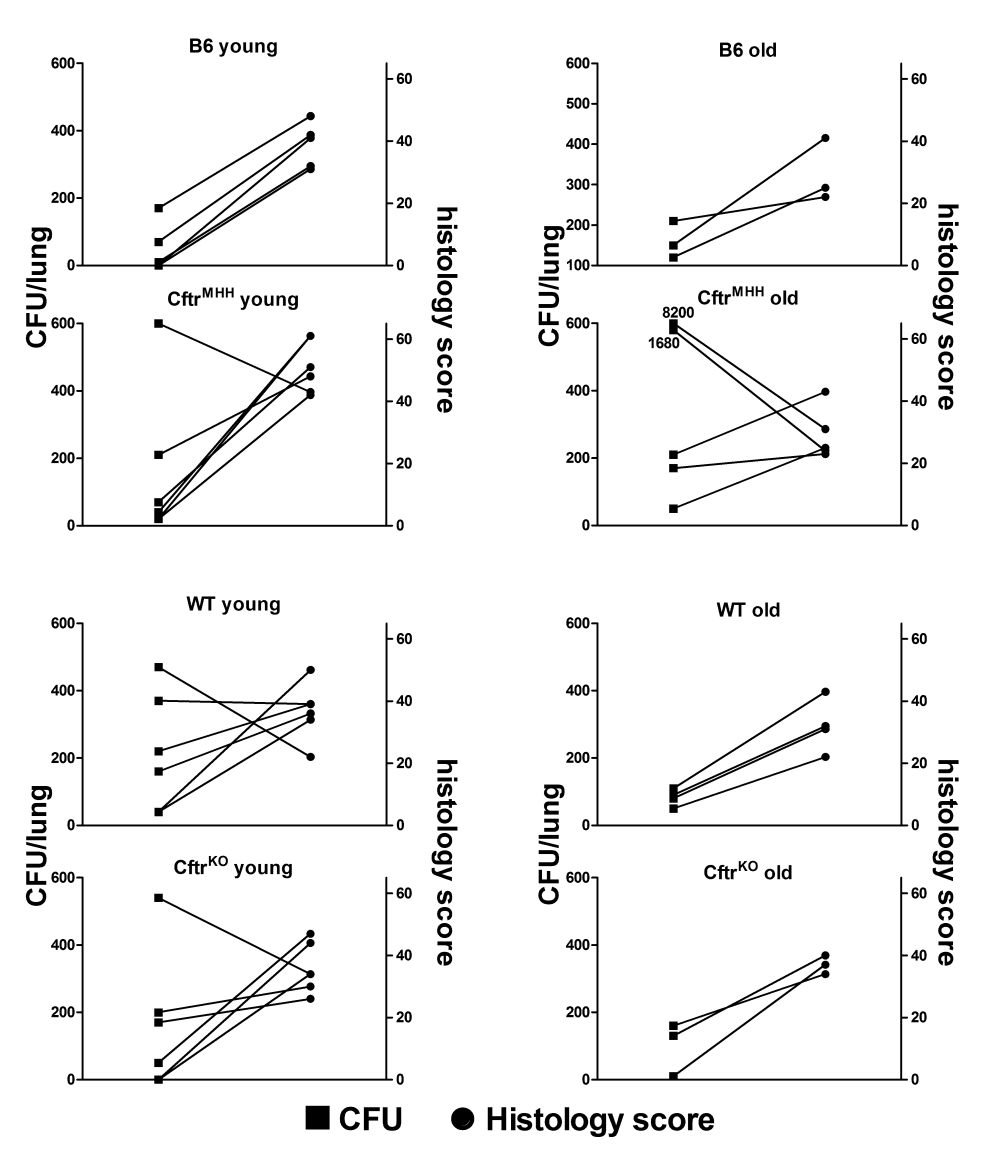
**Bacterial load and inflammation**. Differentiated by age and genotype, the graphs depict the histology inflammation score of the right lung (circles) and the number of viable bacteria recovered from the left lung (squares) at 48 hours after intratracheal instillation of *P. aeruginosa *TBCF10839.

Forty-eight hours after challenge the numbers of viable *P. aeruginosa *TBCF10839 in the murine lungs ranged from zero to a maximum of 8.2 × 10^3 ^CFU (Figure 6). The mice had cleared 99% or more of the initial inoculum. No differences could be observed between young and old and between CF and non-CF mice.

**Figure 7:**
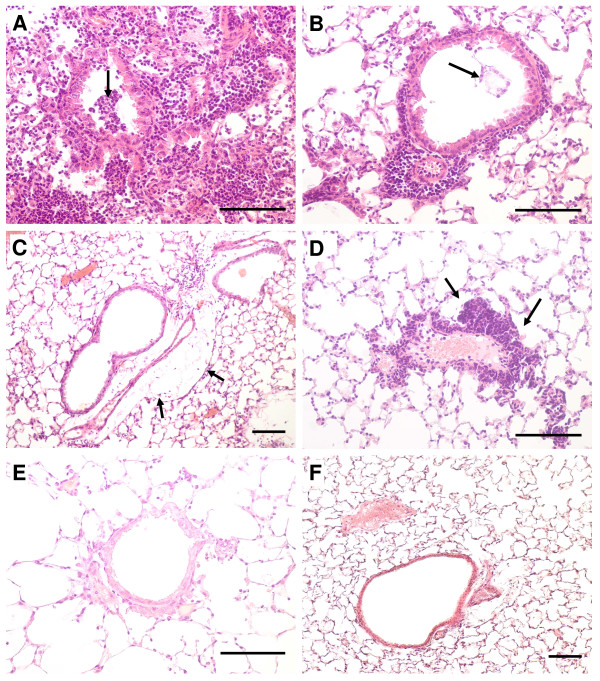
**Lung pathohistology photomicrographs**. Representative examples of the stained specimen slides show the inflammation in the murine lungs 48 h after an intratracheal infection with 6.0 × 10^5 ^CFU of *P. aeruginosa*. Slides A-E represent different degrees of inflammation as they were assessed in the semi-quantitative pathohistological score; control (F). Hematoxilin-eosin staining; scale bar: 100 μm. A) Profound purulent pneumonia in an old FABP WT mouse, massive inflammatory infiltrates within the alveolae and intrabronchiolar (arrow), B) Old FABP WT mouse showing moderate inflammation with peri- and minor intrabronchiolar (arrowhead) leucocyte accumulation, C) Peribronchiolar and perivascular inflammation accompanied by inflammatory edema (arrow) in a young *Cftr^MHH ^*mouse D) Old *Cftr^KO ^*mouse: Strong leucocyte accumulation (arrow) around a lung vessel, E) Only faint peribronchiolar inflammation in an old *Cftr^MHH ^*F) Control: normal lung parenchyma.

Examination of lung histology (Figure 7) revealed most signs of inflammation in the lung parenchyma such as alveolar histiocytosis, PMNs in the alveoli and partially even necrosis of the alveolar septae. In the most strongly affected animals approximately 20-30% of the tissue showed signs of an acute catarrhalic-suppurating alveolar pneumonia. Inflammatory cell infiltrates of bronchi and perivascular edema were seen in less than 10% of examined sections.

**Figure 8:**
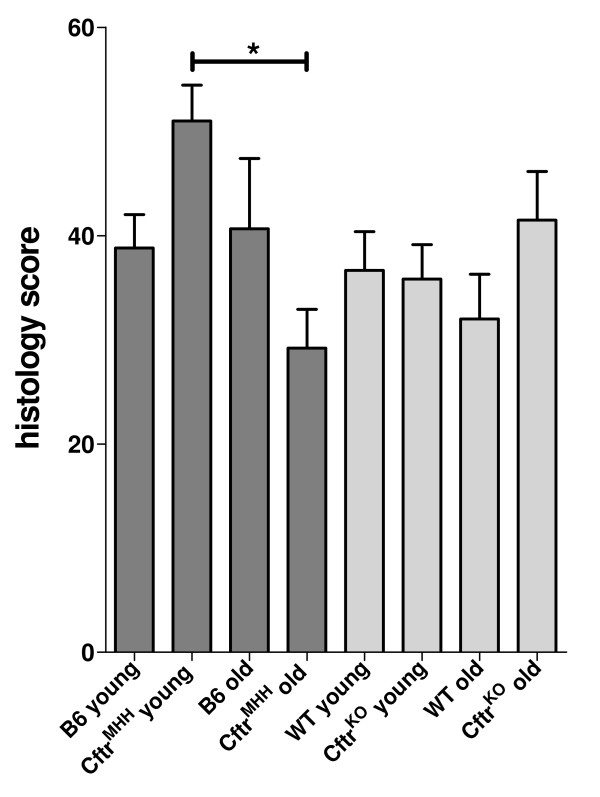
**Mean histology scores of lung inflammation 48 hours after intratracheal instillation of *P. aeruginosa***. The bars show the mean and the standard error of the mean. *, *P *< 0.05; Kruskall-Wallis rank test and Dunn's multiple comparison test of the individual scores.

Among the different animal groups the young *Cftr^MHH ^*mice exhibited a significantly higher inflammation than the old *Cftr^MHH ^*mice in the lung parenchyma, but not in other lung compartments. Correspondingly young *Cftr^MHH ^*mice had the highest mean histology score (Figure 8). At the chosen endpoint of 48 hours the number of viable bacteria in the lungs did not significantly correlate with the severity of airway inflammation classified by the histology score (Figure 6).

## Discussion

This study shows for the first time lung function data of CF mice and demonstrates the impact of age, *cftr *genotype, genetic background and an acute airway infection with *P. aeruginosa *on lung function. Lung function measurements in uninfected CF and non-CF mice showed general trends for the investigated age groups. Tidal volume increased slightly with increased body weight. Respiratory rate decreased as mice breathe slower, which is also observed in the time required for one breath. In general the flows also decreased concordant with the slower respiration. CF and wild type mice had approximately the same tidal volume. However, when further lung function parameters were taken into account, it could be observed that the tidal volume levels of the CF mice were only achieved through faster breathing as characterized by the times for breathing and the respiratory rate. Interestingly these variations between CF and non-CF mice of the same genetic background did not withstand under *P. aeruginosa *airway infection.

Chronic airway infections with *P. aeruginosa *contribute substantially to morbidity and mortality in individuals with CF [33,34]. Numerous infection models have been established in rodents to mimick the *P. aeruginosa *infection in CF, but only the rather artificial bead models with encapsulated *P. aeruginosa *partially succeeded to mimick bacterial persistence in lungs that is typical for human CF airways [35-37]. No chronic *P. aeruginosa *infection model has yet been established in CF mice [10,14,15]. This fact may be ascribed to differences in lung morphology or lung physiology such as the lack of submucosal glands in the lower conducting airways of the mouse or to the endogeneous expression of alternative chloride channels in murine lungs that may at least in part rescue loss-of-function Cftr [11,38]. Although the former argument is probably true and hence calls for alternative infection models in the recently developed CF pigs and CF ferrets [39-45], the latter argument is less convincing because we meanwhile know of many CF patients who express residual amounts of CFTR or alternative ion conductances and still become chronically colonized with *P. aeruginosa *in their airways [46,47]. Hence we propose that we should revisit the CF mouse infection models and try to pinpoint concordant and discordant mechanisms that are operating in CF mice and CF patients.

Only recently a link between *Cftr *genotype and the airway infection with *P. aeruginosa *in mice could be established. CF mice elder than 16 weeks became susceptible to airway colonization with *P. aeruginosa *when infected by the intranasal route [19]. This phenotype was associated with an age-dependent accumulation of ceramide in airway epithelial cells. When ceramide accumulation was prevented by pharmacological or genetic means, the CF mice lost their increased susceptibility to colonization with *P. aeruginosa*.

For the present study we selected exactly the same CF mouse lines and their congenic or transgenic controls, but inoculated *P. aeruginosa *by intratracheal instillation [23]. The congenic B6 mice showed more pronounced symptoms of acute infection than the transgenic mice, but non-CF and CF animals with the same genetic background behaved more or less similar. The old CF mice were not more susceptible to *P. aeruginosa *infection than their age-matched wild type controls.

**Figure 9:**
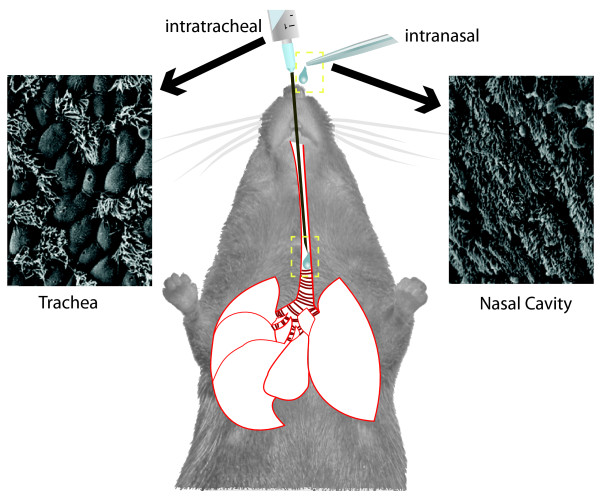
**Experimental lung infection - Impact of infection route**. The sketch highlights the differential experimental set-up for intranasal and intratracheal infection in mice. Compared to an intranasal infection route the intratracheal instillation bypasses the initial host immune response conducted by the respiratory epithelium of the upper airways and deposits more bacteria into the distal airways. The basic defect in CF impairs the mucociliary clearance [48]. Ciliated respiratory epithelium is abundant in the upper and conducting airways, decreasing with the increasing branching of the airways. Correspondingly mucociliary clearance plays a prominent role for the elimination of the pathogen from these compartments, but not from the alveolar space. Please note the differential abundance of respiratory epithelium in the upper airways (right photograph) and the lower conducting airways of mice (left photograph).

Since the present and the previous studies primarily differ in their mode of bacterial infection (Figure 9), we hypothesize that the divergent infection routes explain the inconsistent outcome of the two studies of how and to which extent *P. aeruginosa *colonizes the airways of old CF mice. The intranasal route deposits the bacteria in the upper airways from which they spread to the intestine and the lower airways. In contrast the intratracheal route inoculates the lower airways, preferentially the smaller conducting airways [23]. The intratracheal instillation thus bypasses the initial steps of any lung infection, i.e. the colonization of the upper airways and the largest lower airways with the (opportunistic) pathogen (Figure 9).

We have previously investigated the CF phenotype of trachea and upper airways of *Cftr^MHH ^*mice [21,25]. Like in humans, the nasal epithelium of the CF mice exhibited the basic defect of Na^+ ^hyperabsorption and Cl^- ^hyposecretion [25] and the trachea had accumulated ceramide [21]. The basic defect of perturbed electrolyte transport across the apical epithelial membrane translates into airway surface dehydration and impaired mucociliary clearance [48], and we have indeed measured impaired clearance in *Cftr^MHH ^*mice [49]. Correspondingly, if CF mice were exposed intranasally with *P. aeruginosa *the bacterial clearance did not work efficiently in the upper airways of CF mice and the bacterial load increased within the first hours [19]. In contrast, if the murine lung was inoculated with *P. aeruginosa *by intratracheal instillation, the bacterial clearance from the upper airways and the large conducting lower airways was bypassed and the host response to the intratracheal infection route was indistinguishable between congenic CF and non-CF mice. Thus we would like to conclude that the CF condition undermines the first barrier of host defense, i.e. bacterial clearance, but does not compromise the subsequent host responses. This conclusion fits with our current knowledge of how the basic defect in CF patients predisposes to infection in conducting airways [40,48,50,51]: CFTR-deficiency impairs ciliary clearance and slows down mucus transport thus facilitating bacterial colonization, particularly if the airways are injured by an acute viral infection [52].

## Conclusions

Hence, the bottom-line of our previous and present studies is that CF mice are suited to investigate of how the basic defect translates into an increased susceptibility to airway colonization with *P. aeruginosa*. The first line of host defense, i.e. the removal of bacteria from the airways by mucociliary clearance is deficient in CF mice. However, the subsequent steps of host-pathogen interaction during an acute infection with *P. aeruginosa *are not compromised in CF mice. In other words, CF mice are appropriate models to study the very early host defense mechanisms.

## Competing interests

The authors declare that they have no competing interests.

## Authors' contributions

AM designed and carried out the infection experiments and drafted the manuscript. FW participated in the infection experiments, did the spirometry measurements and performed the statistical analysis. TKM participated in the infection experiments. DW supervised the breeding of the utilized mouse models and did the genotyping of the *Cftr^KO ^*mice and their respective littermates. UB was responsible for the make-up of the spirometry unit. EG assisted in the concept of the study. BT conceived the study, participated in its design and coordination and in the writing of the manuscript. All authors read and approved the final manuscript.

## Supplementary Material

Additional file 1 **Longitudinal values for spirometric parameters**. The comprehensive table shows the courses of each single spirometric parameter. Significant differences are marked in grey. Click here for file
